# Impact of Feeding Level and Multi-Nutrient Blocks with Polyherbals on Weight Changes and Greenhouse Gas Emissions in Lambs

**DOI:** 10.3390/ani15172541

**Published:** 2025-08-29

**Authors:** Nallely Sánchez-López, Germán David Mendoza-Martínez, María Eugenia de la Torre-Hernández, Pedro Abel Hernández-García, Cesar Díaz-Galván, Gilberto Carlos Ortega-Navarro, Mariela Hada Fuentes Ponce, Abel Jaime Leal-González, Santiago López Ridaura, Jelle Van Loon

**Affiliations:** 1 SECIHTI Programa de Investigadoras e Investigadores por México, Universidad Autónoma Metropolitana Xochimilco, Mexico 04960, Mexico; nsanchezl@correo.xoc.uam.mx (N.S.-L.); mdelatorre@correo.xoc.uam.mx (M.E.d.l.T.-H.); 2 División de Ciencias Biológicas y de la Salud, Departamento de Producción Agrícola, Animal Universidad Autónoma Metropolitana Xochimilco, Mexico 04960, Mexico; gmendoza@correo.xoc.uam.mx (G.D.M.-M.); cesarwardi14@gmail.com (C.D.-G.); gilbertonavarro27@hotmail.com (G.C.O.-N.); mfponce@correo.xoc.uam.mx (M.H.F.P.); 3 Centro Universitario Amecameca, Universidad Autónoma del Estado de México, Toluca 50110, Mexico; 4 International and Wheat Improvement Center (CIMMYT), El Batan 56237, Mexico; a.leal@cgiar.org (A.J.L.-G.); s.l.ridaura@cgiar.org (S.L.R.); j.vanloon@cgiar.org (J.V.L.)

**Keywords:** multi-nutrient blocks, lambs, enteric methane emission, polyherbals, maintenance, small livestock production

## Abstract

In small-scale livestock production systems, low-quality diets constrain animal performance and increase enteric emissions, but both these impacts can be remediated using optimized feeding strategies. An experiment was conducted with lambs fed at two levels—maintenance (MN) and growth (GR)—using multi-nutrient blocks formulated with different concentrations of polyherbal nutraceuticals to compare the lambs’ reactions in terms of their productive performance and estimated enteric methane emissions. Thirty-two lambs were fed at two feeding levels—(a) maintenance (MN) at 9% CP and 1.85 Mcal ME/kg DM and (b) growth (GR) at 13.24% CP and 2.15 Mcal ME/kg DM)—and did or did not have access to MBs with different polyherbal percentages (BioCholine^®^, OptiLysine^®^, and OptiMethione^®^ (0:0:0, 3:0:0, 3:0.75:0.25)). No interactions between the feeding level and supplementation were detected. Lambs fed at the MN level showed lower productive indicators (*p* < 0.001) than those fed at the GR level, with a lower dry matter intake (DMI, 512 vs. 1009 g/d), MB consumption (61 vs. 84 g/d), and daily weight gain (26 vs. 187 g/d), resulting in lower enteric methane emissions (8.74 vs. 18.18 g CH_4_ /d) and a lower emission intensity (15.25 vs. 16.55 CH_4_ g/kg DM). Supplementation with MBs improved the average daily weight gain (ADG) (*p* < 0.001) at the GR level, but no differences were detected at the MN level. However, lambs in the control group lost weight (−20 g/d) and those supplemented gained weight (g/d), with increases of 49 (0:0:0), 25 (3:0:0), and 52 (3:0.75:0.25). The highest ADG for lambs in the GR group was observed with MBs containing all three polyherbals (215a, 3:0.75:0.25), an intermediate ADG was seen with MBs without herbals or with Biocholine (200.75ab, 0:0:0; 198ab, 3:0:0), and the lowest ADG was observed with no MBs (134c g/d). The use of MBs reduces the time to reach market weight by 265 days, resulting in a 50% reduction in the enteric methane emissions per product (animal by animal), making multi-nutrient blocks a viable option to improve production indicators and reduce enteric methane emissions.

## Simple Summary

The nutritional quality of diets is a key factor affecting both animal performance and methane emissions in small-scale livestock production systems. An experiment was conducted that involved feeding lambs multi-nutrient blocks (MBs) that included nutraceutical herbal products at maintenance (MN) and growth (GR) levels. Lambs in the MN group exhibited lower performance indicators and lower methane emissions compared to those in the GR group. Lambs in the GR group that consumed MBs showed an increase in their daily weight gain. Using MBs improves weight gain and reduces the methane emissions per animal.

## 1. Introduction

Sheep farming in Mexico is carried out within specialized or family farming systems, with family systems representing around 75% of the national sheep population [1,2]. Within this production system, the application of formulated rations designed to meet the animals’ complete nutritional requirements (crude protein, energy, vitamins, and minerals) is uncommon, which results in suboptimal animal performance [3] and, in turn, increased enteric methane emissions per unit of animal product or per unit of feed consumed [4] compared to those in specialized systems.

An alternative way to complement the diet of animals in family farming systems with nutrients is to use nutrient-dense blocks as supplements; these have been reported to elicit favorable responses in ruminants [5,6,7], including those on low-quality diets [8,9]. However, the response related to gains in animal productivity has been variable depending on the feeding system [10,11], where the basal rations have had a determining impact on the productive indicators.

There are commercial nutrient blocks with different formulations available [12,13], as well as blocks formulated with local resources [7,11,14], and both constitute a strategy to improve ruminant production [15]. Multi-nutrient blocks (MBs) stand out for their high protein content and their supply of macro- and micro-elements in various formulations. Some incorporate nutraceutical additives that, in addition to providing nutrients, may help reduce the enteric methane emissions [16] and, depending on their bioactive compounds, may also contribute to lowering parasitic loads [17].

Block formulations may include polyherbal additives to take advantage of the identified nutrients [18,19,20], as well as the contribution of secondary metabolites, which may help reduce enteric methane production [21].

Therefore, the objective of this experiment was to evaluate the response of lambs to multi-nutrient blocks containing herbal products with nutraceutical properties under two nutritional levels (maintenance and growth), focusing on their growth performance and enteric methane emissions. It was hypothesized that formulating blocks with nutraceutical additives would improve lambs’ growth indicators and reduce the enteric methane emissions per lamb produced.

## 2. Materials and Methods

This study followed the protocol and procedures presented, which were approved by the Committee for Care and Use of Experimental Animals of the Autonomous University of the State of Mexico, Campus Amecameca, which approved the procedures under Protocol Number 1, 2025. The experiment was divided into two periods with durations of 30 and 50 days for the first and second periods, respectively, and used 32 lambs (initial weight of 15.63 ± 2.86, Katahdin × Crossbreeds), which were given vitamins (Vigantol vitamin A, D, and E from Bayer, 2 mL) and dewormed (Closantel, 5 mg/kg of BW) at the beginning of the experiment. Three kinds of multi-nutrient blocks were formulated, incorporating the polyherbal mixtures BioCholine^®^, OptiLysine^®^, and OptiMethione^®^ (Nuproxa Mexico, Swiss Nuproxa Group, Etoy, Switzerland; Indian Herbs Co., Saharanpur, India) at different mixture percentages, as shown in Table 1. The lambs were fed individually in consecutive periods at two feeding levels: maintenance and growth (Table 2). From the samples for each period, dry matter, ash, crude protein, and ether extract were collected and analyzed using AOAC procedures [22], and neutral detergent fiber (NDF) and acid detergent fiber (ADF) were collected and analyzed using the methods of Van Soest et al. [23].

Lambs were randomly assigned to four groups of eight lambs each undergoing predefined treatments, consisting of a control group without supplementation and three groups with access to the three blocks formulated with different percentages of polyherbals (phosphatidylcholine/lysine/methionine) selected for their nutraceutical and bypass properties [18,19,20]. The lambs had free access to the rationed feed and water; feed was offered at 8:00 h and 15:00 h. Their dry matter intake (DMI) and block intake were recorded daily. The lambs were weighed on two consecutive days at the beginning of each period (i.e., days 30 and 50, respectively) to evaluate their weight changes and feed conversion.

The enteric methane was estimated using mechanistic equations based on the fermentable carbohydrate intake [24], calculating the moles of hexose fermented ruminally from the fermentable carbohydrate intake in grams divided by the molecular weight of glucose [25]. The non-fiber carbohydrates in the cellular contents were considered 98% digestible. The in vitro dry matter digestibility (IVDMD) was used to estimate the NDF digestion, and then the true NDF digested was obtained through a correction using the metabolic fecal N and lipids (12.9%) as follows [26]:

Fermentable CH_2_O intake g/d = {[(IVDMD %/100) × NDF (%) × 1.129] + 0.98 × (Non fiber carbohydrates %)]} × DM intake g/d

Moles of hexose fermented = (Fermentable CH_2_O intake g/d)/162

The moles of methane (CH_4_) and carbon dioxide (CO_2_) were estimated using an in vitro gas technique, modifying the methodology proposed by Menke and Steingass [27] to estimate the CH_4_ and CO_2_ from the maximum gas volume [28].

**Table 2:** Formulation of basal experimental rations and chemical composition (dry basis).

	Maintenance	Growth
Corn stover, %	71.0	50.0
Cracked corn, %	21.0	34.0
Soybean meal, %	8.0	16.0
Total	100	100
Chemical composition		
Dry matter, %	89.74	88.92
Neutral detergent fiber, %	59.18	49.17
Crude protein, %	9.00	13.24
Ash, %	6.32	5.58
Ether extract, %	2.25	2.46
Metabolizable energy, Mcal/kg ^a^	1.85	2.15

^a^ Metabolizable energy estimated based on information provided by NRC [29].

Thermos bottles were pre-heated with hot water at 39 °C, which was discarded prior to filling them with the ruminal fluid. The rumen inoculum was filtered through four layers of gauze before being collected in the thermos and then immediately transported to the laboratory. The rumen inoculum was mixed with a reduced mineral solution at a 1:9 (*v*/*v*) ratio. The mineral solution (per liter) contained 0.45 g K_2_HPO_4_, 0.45 g KH_2_PO_4_, 0.45 g (NH_4_)_2_SO_4_, 0.90 g NaCl, 0.18 g MgSO_4_, 0.12 g CaCl_2_, and 4.00 g Na_2_CO_3_. This solution was reduced with 20 mL/L of a reducing mixture composed of 0.2 g Na_2_S and 0.2 g L-cysteine, dissolved in a 0.8 mL/L NaOH solution. To confirm the reduction, two drops of 0.1% (*w*/*v*) resazurin were added as a redox indicator. Flasks containing only the inoculum and medium (without substrates) were used as blanks to correct for gas production. After 24 h of incubation, the residues from flasks from each treatment filtered through Waltham No. 541 filter paper. The retained residues were then weighed to estimate the in vitro dry matter digestibility. The flasks were incubated in a water bath (39 °C), and the gas pressure was monitored and recorded at 3, 6, 9, 12, 24, 36, and 48 h using a manual manometer (Amphenol SSI Technologies) [30], and the accumulated gas volume was recorded using a 60 mL graduated hypodermic syringe. The gas trapped in the syringe was transferred by injection to another hermetically closed flask with 40 mL of a sodium hydroxide solution (1 M KOH) to fix the carbon dioxide, forming potassium bicarbonate [4,31], and we estimated the methane production using the difference. Methane estimation using an in vitro method has been previously validated [32].

The methane and carbon dioxide were expressed per unit of the intake (grams/kg of the DMI), per kg of the average daily gain (grams per g/kg of the ADG) [24], and per kg of lamb produced [4], excluding information from lambs that lost weight in the maintenance feed level group.

### 2.1. Enteric Methane and Carbon Dioxide Estimations

As mentioned above, the proportions of methane and carbon dioxide were estimated using an in vitro gas technique. Five hundred mg of formulated dietary treatments were incubated with a ruminal fluid inoculum obtained from two sheep. To validate the methane estimates obtained using the previously described mechanistic equations, 60 pieces of in vivo data from the sheep, measured using the SF_6_ tracer technique and respiration chambers, were selected. These records included information on the diet’s chemical composition, in vivo digestibility, body weight, and dry matter intake and were extracted from the supplementary material in Clauss et al. [33]. The methane emissions were then estimated using the equations and compared with the observed values.

### 2.2. Statistical Analysis

The data normality was tested (Shapiro–Wilk), and the results were analyzed using a completely randomized design with four dietary treatments (n = 8 sheep) with a repeated measurements mixed model:

*Y_ijk_* = *μ* + *T_i_* + *P_j_* + (*T×P*)*_ij_* +*A_k_* + *ε_ijk_* (1)

where

*Y*_*ijk*_: response variable representing the observed value at time (period) *j* for treatment *i* and subject (lamb) *k*.

*μ*: overall mean.

*T*_*i*_: fixed effect of the dietary treatment.

*P_j_:* fixed effect of the period (maintenance and growth in the experiment).

(*T*×*P*) _*ij*_: interaction between the dietary treatment and period (maintenance or growth).

A_*k*_: random animal effect.

*ε*_*ijk*_: residual error.

The means were compared using the Tukey test. All statistical analyses were performed with SAS (v9.4, SAS, Cary, NC, USA) on demand for academics.

The estimated values for the enteric methane (g/d) were compared with the observed values using a Tukey test (alpha = 0.05; n = 60). A linear regression was performed between the estimated and predicted values, comparing the slope to unity and the intercept to zero [34].

## 3. Results

### Validation of Methane Estimation

**Figure 1:**
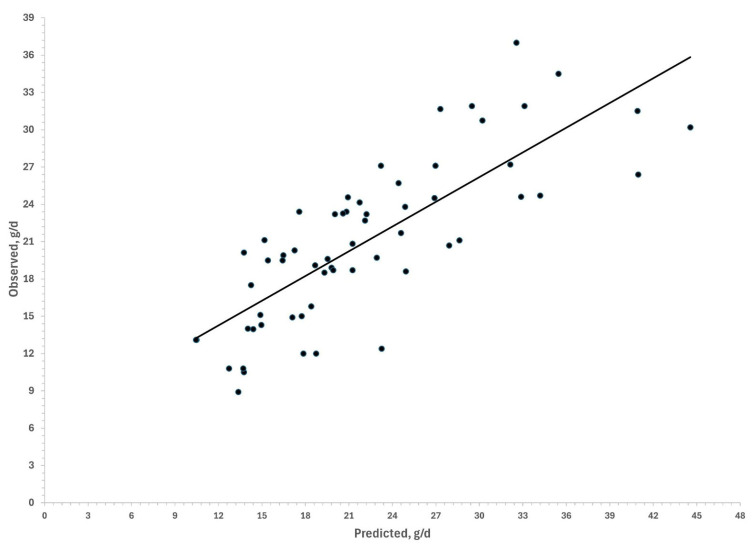
Estimation of observed values using mechanistic equations (Y = 6.295 ± 3.165 + 0.663 ± 0.135 X; n = 60, r^2^ = 0.62; *p* < 0.0001).

Estimation of the enteric methane using mechanistic equations resulted in values similar to those measured in vivo (Table 3), with significant predictions (Observed CH_4_ = 6.295 ± 3.165 + 0.663 ± 0.135 Predicted CH_4_; r^2^ = 0.62; *p* < 0.0001; Figure 1).

No interactions were detected between the feeding level (period) and supplementation type (period × treatment); therefore, the main effects are presented. Lambs fed at maintenance showed lower productive indicators (*p* < 0.001) than those fed at growth, with a lower dry matter intake and consumption of multi-nutrient blocks, resulting in lower daily weight gains and lower daily enteric methane emissions. However, the daily methane emissions per kg of lamb were 3.4 times higher when the lambs were fed at above the maintenance level (Table 4).

Supplementation with multi-nutrient blocks increased the ADG: the highest gains were found for MBs with all three polyherbals (3:0.75:0.25), intermediate gains for MBs without herbals (0:0:0) or with BioCholine (3:0:0), and the lowest gains for no MBs. The daily methane emissions were not different with MBs, but when expressed per unit of the lambs produced, lambs without access to MBs emitted 1.83 times more methane than those with access to the MB supplement (Table 5).

**Figure 2:**
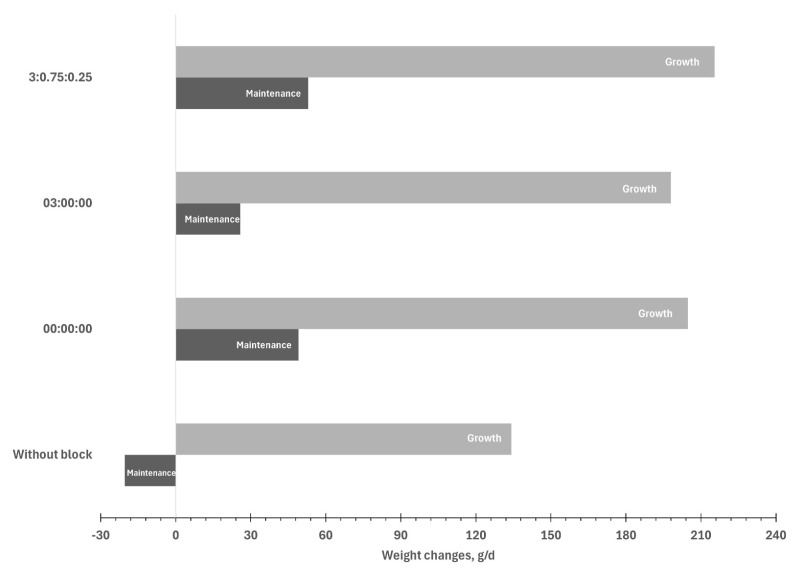
Average daily gain in lambs fed at maintenance and growth levels with and without multi-nutritional block supplementation with different percentages of polyherbal inclusion (phosphatidylcholine/lysine/methionine).

Figure 2 shows the weight changes by the treatment: lambs receiving maintenance-level rations without MB supplementation lost weight (−20 g/d), but when receiving the multi-nutrient blocks, all the lambs gained weight.

## 4. Discussion

### 4.1. Validation of Methane Estimates with Mechanistic Equations

It is recognized that the best methods for estimating the amount of greenhouse gases (GHGs) involve chambers/respiration chambers and the SF6 technique; however, this is limited by the availability of equipment and economic resources [35], so it is important to have reliable equations for estimating the enteric methane that allow for estimates of changes based on the feed and supplement consumption. Accurate estimates of the methane production by ruminants in different contexts are essential to developing mitigation strategies under different conditions [36]. Mechanistic equations have previously been used to estimate the impact of dietary changes in lambs [24,25] and cattle [37,38]. Mechanistic models enable the estimation of the impacts of dietary changes and the evaluation of mitigation strategies and are preferable to models based on empirical equations [39]. In a review of the published models [40], it was noted that all models have limitations and uncertainties and that there is no ideal model. However, it is necessary to validate individual methods, compare methods, and develop calibration and standardization protocols for existing methods.

### 4.2. Lamb Performance and Methane Emissions

As expected, differences in the nutrient intake between the lambs fed maintenance rations and those receiving growth rations had a significant impact on the animal performance [41]. The composition of the diets reflected standard feeding practices commonly used by small-scale producers in Mexico [2], characterized by a low protein content, a limiting nutrient for growth when supplied at below the recommended levels [42,43]. However, the observed response depended not only on the total intake of digestible nutrients [44] but also on the characteristics and proportions of digestible, potentially digestible, and indigestible NDF in the rations [24]. Based on the lamb performance determined using energetic equations for lambs [45], we estimate that at the maintenance level, the dietary net maintenance energy (NEm) was 1.076 and the net energy gain (NEg) was 0.533 Mcal/kg DM, while at the growth level they increased notably (1.391 NEm and 0.810 Mcal/kg NEg).

Most family sheep producers in Mexico do not use supplements, as they base their feeding on free grazing, a practice common in many other regions of the Global South [46]. Although the DM intake was not modified with the MBs, the protein intake increased by 30%, the net maintenance energy by 37.5%, and the net energy gain by 71.4% compared to those of lambs on unsupplemented diets (Mcal/kg DM: 1.329 NEm and 0.756 NEg vs. 0.984 NEm and 0.453 NEg). Multi-nutrient blocks allow for a higher intake of digestible nutrients [47] and have also been used to reduce the use of feed concentrates [13].

The inclusion of different herbal nutraceuticals in the MBs did not modify the DM or nutrient block intake; however, the better response in terms of lamb productivity with all three polyherbals could be explained by the higher intake of limiting nutrients (phosphatidylcholine, lysine, and methionine). The NRC [29] has not established requirements for metabolizable lysine and methionine in sheep; however, ruminally protected amino acids have improved ovine growth and health, as well as having other beneficial effects [48].

The results regarding the supplementation of rumen-protected lysine and methionine in lambs have been inconsistent, potentially due to differences in the amino acid ratios used. In this study, herbal amino acids were included at a 3:1 lysine-to-methionine ratio, which is recommended for dairy cattle [38]. Al-Badri et al. [49] reported that lysine and methionine, alone or combined in a 1:1 ratio, improved performance compared to unsupplemented diets. Liu et al. [50] found that the average daily gain (ADG) improved in lambs with a lysine/methionine ratio of 4:1 but declined when the ratio increased to 7:1. These findings suggest that further research is needed to determine the optimal amino acid duodenal flow for supplementation in growing lambs [51].

Herbal products, in addition to providing nutrients, also offer various secondary metabolites that can influence different metabolic pathways [21,52], enhance the methylation status [53], reduce ruminal methane emissions [54], and provide antioxidants [55]. Among the various secondary metabolites, some have been reported to improve the meat [52,56] and milk quality [57]. It has been suggested that BioCholine can meet sheep’s choline needs; however, the NRC [29] has not established choline requirements in sheep. Nevertheless, it is recognized that protected choline or herbal BioCholine can improve an animal’s response due to increased energy production [18], which would explain the difference between the outcomes when using MBs with and MBs without BioCholine or herbal supplements (Figure 1).

Feed management, diet formulation, and rumen manipulation strategies have been recognized as the main animal GHG mitigation strategies [58]. Feeding lambs on maintenance rations increased the methane emitted per kg of lamb produced 2.2 times. Reducing the number of days to slaughter due to improved growth directly impacts the total gas emissions [59]. Additionally, it has been recognized that enhancing the productivity of lower-producing animals has a significant impact on methane emission reduction [60]. Lambs that did not receive MBs produced 1.8 times more enteric methane per kg of lamb. MBs can be a way to include additives with different effects that contribute to reducing the enteric methane, similarly to feed plant additives and natural and synthetic methanogenesis inhibitors [61]. It is essential to generate information that can be applied to family livestock systems, as these represent the main source of income and a community resilience component for around 200 million smallholder families in Asia, Africa, and Latin America [1]. The results of this study show that in low-productivity systems, the GHG emissions are significantly higher than in systems with animals that are better fed, and that they could be significantly reduced. Moreover, feeding lambs maintenance rations with MBs could double the production of sheep meat in these systems, contributing to the growing demand for food in a sustainable way and reducing GHG emissions; the absence of MBs doubles the time for lambs to reach market weight (from 500 to 234 days). These findings align with SDG 13 (Climate Action) and SDG 2 (Zero Hunger) by promoting climate-smart livestock production in marginal systems.

## 5. Conclusions

In low-quality diets, feeding maintenance diets and supplementing these with multi-nutrient blocks improves lambs’ performance and reduces the enteric methane emissions per kg of sheep produced. Block formulations can include herbal additives to improve the lamb performance indicators.

## Figures and Tables

**Table 1:** Formulation of experimental multi-nutrient blocks (MBs) with different polyherbal nutraceutical compositions (dry basis).

	Phosphatidylcholine/lysine/methionine		
	(0:0:0)	(3:0:0)	(3:0.75:0.25)
Molasses	40	40	40
Corn stover	13	13	13
Soybean meal	10	5	5
Urea	10	10	10
Ground corn	9.85	9	9
Mineral premix ^a^	6	6	6
Cement	5.15	5.85	4.85
Lime	5	4.4	4
Salt	1	0	0
Sodium sulfate	0	1	1
Chelated minerals ^b^	0	0.15	0.15
Sodium propionate	0	1	1
Sodium hexametaphosphate	0	2	2
BioCholine	0	3	3
OptiMethionine	0	0	0.25
OptiLysine	0	0	0.75
TOTAL	100	100	100
Chemical composition			
Dry matter, %	84.66	85.06	86.87
Ash, %	24.4	26.84	25.08
Crude protein, %	36.96	34.39	35.07
Neutral detergent fiber, %	13.97	12.96	13.0
Ether extract, %	0.77	0.66	0.55

^a^ Vitasal Engorda Ovino Plus: 270 g Ca, 30 g P, 7.5 g Mg, 65.5 g Na, 100 Cl, 0.5 g K, 42 mg S, 2000 mg Lasolacid, 2000 mg Mn, 3000 mg Zn, 20 mg Se, 15 mg Co, 35,000 UI vitamin A, 150,000 UI vitamin D, and 150 UI vitamin E. ^b^ Ovi3 ways: 590 mg Se, 990 mg Cr, 1500 mg Cu, 3000 mg Fe, 3000 Zn, 3000 Mn, 30 mg Co, 30 mg Y, 400 UI vitamin E, and 1 × 1012 UFC/kg Saccharomyces cerevisiae.

**Table 3:** Validation of enteric methane estimation using mechanistic equations (n = 60) with observed values.

	Methane, g/d
Observed	20.98 ^a^
Mechanistic equations	22.15 ^a^
SEM	0.7632

^a^: When superscripts differ within a row, it indicates significant differences (*p* ≤ 0.05) between the treatments. SEM: Standard error of the mean.

**Table 4:** Main effects of feeding levels on lambs’ performance and enteric methane emissions.

	Feeding Level			
	Maintenance	Growth	SEM	*p*-Value
Initial BW, kg	15.63	16.87	0.578	0.13
Final BW, kg	16.43 ^b^	26.23 ^a^	0.835	0.0001
Dry matter intake, g/d	512.8 ^b^	1009.1 ^a^	37.404	0.0001
Block intake, g/d	61.3 ^b^	84.2 ^a^	5.9102	0.0083
ADG, g	26.87 ^b^	187.12 ^a^	9.1097	0.0001
Estimated methane				
CH_4_, g/d	8.74 ^b^	18.18 ^a^	1.320	0.0001
CH_4_, g/kg DMI	15.25 ^b^	16.65 ^a^	0.436	0.0001
CH_4_, g/kg PV^0.75^	1.08 ^b^	1.81 ^a^	0.096	0.0001
CH_4_/kg of lamb, g	231.14	105.47	15.519	0.0002

^a, b^: When superscripts differ within a row, it indicates significant differences (*p* ≤ 0.05) between the treatments. SEM: Standard error of the mean.

**Table 5:** Main effects of multi-nutrient block supplementation and polyherbal inclusion on lambs’ performance and enteric methane emissions.

	Polyherbal % Included in Multi-Nutrient Block (Phosphatidylcholine/lysine/methionine)					
	Control	(0:0:0)	(3:0:0)	(3:0.75:0.25)	SEM	*p*-Value
Initial BW, kg	16.58	16.35	16.09	15.99	0.8176	0.95
Final BW, kg	19.63	22.04	21.50	22.18	1.1819	0.40
Dry matter intake, g/d	779.5	728.8	728.4	807.11	52.898	0.65
Block intake, g/d	0.0 ^b^	112.4 ^a^	89.6 ^a^	89.03 ^a^	8.3584	0.0001
Average daily gain (ADG), g	56.9 ^b^	123.5 ^a^	113.2 ^a^	134.2 ^a^	18.2194	0.0004
Estimated methane						
CH_4_, g/d	13.33	14.40	13.85	15.31	0.9561	0.52
CH_4_, g/kg DMI	17.10	19.76	19.01	18.96	0.4433	0.70
CH_4_, g/kg PV^0.75^	1.48	1.33	1.34	1.48	0.0418	0.15
CH_4_/kg of lamb produced	388.17	213.47	215.27	207.50	15.5186	0.19

^a, b^: When superscripts differ within a row, it indicates significant differences (*p* ≤ 0.05) between the treatments. SEM: Standard error of the mean.

## Data Availability

The raw data supporting the conclusions of this article will be made available by the authors on request.
